# The HIV-1 reservoir landscape in persistent elite controllers and transient elite controllers

**DOI:** 10.1172/JCI174215

**Published:** 2024-02-20

**Authors:** Carmen Gasca-Capote, Xiaodong Lian, Ce Gao, Isabelle C. Roseto, María Reyes Jiménez-León, Gregory Gladkov, María Inés Camacho-Sojo, Alberto Pérez-Gómez, Isabel Gallego, Luis E. Lopez-Cortes, Sara Bachiller, Joana Vitalle, Mohamed Rafii-El-Idrissi Benhnia, Francisco J. Ostos, Antonio R. Collado-Romacho, Jesús Santos, Rosario Palacios, Cristina Gomez-Ayerbe, Leopoldo Muñoz-Medina, Andrés Ruiz-Sancho, Mario Frias, Antonio Rivero-Juarez, Cristina Roca-Oporto, Carmen Hidalgo-Tenorio, Anna Rull, Julian Olalla, Miguel A. Lopez-Ruz, Francesc Vidal, Consuelo Viladés, Andrea Mastrangelo, Matthias Cavassini, Nuria Espinosa, Matthieu Perreau, Joaquin Peraire, Antonio Rivero, Luis F. López-Cortes, Mathias Lichterfeld, Xu G. Yu, Ezequiel Ruiz-Mateos

**Affiliations:** 1 Institute of Biomedicine of Seville (IBiS), Virgen del Rocio University Hospital, Spanish National Research Council (CSIC), University of Seville, Clinical Unit of Infectious Diseases, Microbiology and Parasitology, Seville, Spain.; 2 Ragon Institute of MGH, MIT and Harvard, Cambridge, Massachusetts, USA.; 3 Infectious Disease Division, Brigham and Women’s Hospital, Boston, Massachusetts, USA.; 4 Clinical Unit of Infectious Diseases and Microbiology, Virgen Macarena University Hospital, Seville, Spain.; 5 Department of Medicine and Microbiology, School of Medicine and; 6 IBiS, Virgen Macarena University Hospital, CSIC, University of Seville, Seville, Spain.; 7 CIBERINFEC, Institute of Health Carlos III (ISCIII), Madrid, Spain.; 8 Department of Medical Biochemistry, Molecular Biology, and Immunology, School of Medicine, University of Seville, Seville, Spain.; 9 Infectious Diseases Unit, Internal Medicine Service, Torrecardenas University Hospital, Almeria, Spain.; 10 Infectious Diseases, Microbiology and Preventive Medicine Unit, Virgen de la Victoria University Hospital, Malaga, Spain.; 11 Unit of Infectious Diseases, San Cecilio University Hospital, Biohealth Research Institute, IBS-Granada, Granada, Spain.; 12 Service of Infectious Diseases, Reina Sofía University Hospital, Maimonides Biomedical Research Institute of Cordoba (IMIBIC), Córdoba University, Cordoba, Spain.; 13 Unit of Infectious Diseases, Virgen de las Nieves University Hospital, Biohealth Research Institute, IBS-Granada, Granada, Spain.; 14 Joan XXIII University Hospital of Tarragona, IISPV, University of Rovira i Virgili, Tarragona, Spain.; 15 Internal Medicine Department, Costa Del Sol Hospital, Marbella, Spain.; 16 Service of Immunology and Allergy, Lausanne University Hospital and; 17 Service of Infectious Diseases, Lausanne University Hospital, University of Lausanne, Lausanne, Switzerland.

**Keywords:** AIDS/HIV, Virology, AIDS vaccine

## Abstract

**BACKGROUND:**

Persistent controllers (PCs) maintain antiretroviral-free HIV-1 control indefinitely over time, while transient controllers (TCs) eventually lose virological control. It is essential to characterize the quality of the HIV reservoir in terms of these phenotypes in order to identify the factors that lead to HIV progression and to open new avenues toward an HIV cure.

**METHODS:**

The characterization of HIV-1 reservoir from peripheral blood mononuclear cells was performed using next-generation sequencing techniques, such as full-length individual and matched integration site proviral sequencing (FLIP-Seq; MIP-Seq).

**RESULTS:**

PCs and TCs, before losing virological control, presented significantly lower total, intact, and defective proviruses compared with those of participants on antiretroviral therapy (ART). No differences were found in total and defective proviruses between PCs and TCs. However, intact provirus levels were lower in PCs compared with TCs; indeed the intact/defective HIV-DNA ratio was significantly higher in TCs. Clonally expanded intact proviruses were found only in PCs and located in centromeric satellite DNA or zinc-finger genes, both associated with heterochromatin features. In contrast, sampled intact proviruses were located in permissive genic euchromatic positions in TCs.

**CONCLUSIONS:**

These results suggest the need for, and can give guidance to, the design of future research to identify a distinct proviral landscape that may be associated with the persistent control of HIV-1 without ART.

**FUNDING:**

Instituto de Salud Carlos III (FI17/00186, FI19/00083, MV20/00057, PI18/01532, PI19/01127 and PI22/01796), Gilead Fellowships (GLD22/00147). NIH grants AI155171, AI116228, AI078799, HL134539, DA047034, MH134823, amfAR ARCHE and the Bill and Melinda Gates Foundation.

## Abstract 2

Before losing virological control, transient controllers present higher levels of intact, non-clonally expanded provirus located in genic regions, compared to persistent controllers.

## Graphical abstract

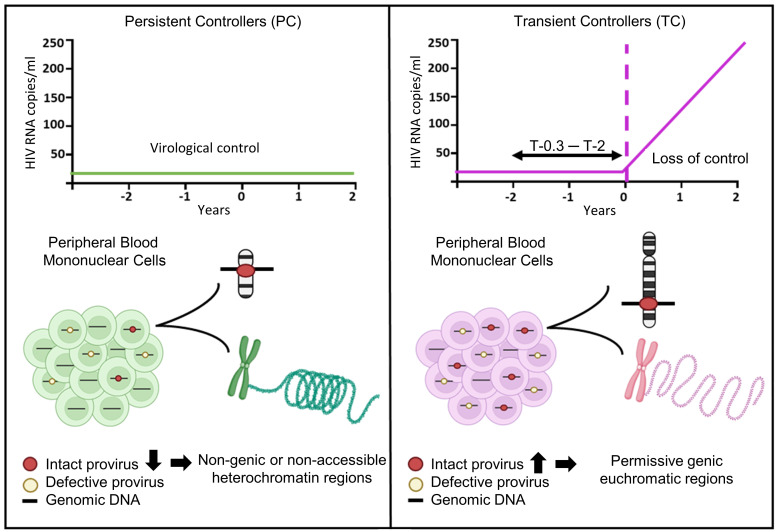

## Introduction

Elite controllers (ECs) represent a small subset of people living with HIV-1 (PLHIV), less than 1%, who can maintain undetectable viral load in the absence of antiretroviral therapy (ART) (1–3). ECs consist of a heterogeneous and dynamic group regarding virological, immunological, and clinical factors (4–7). This has enabled us to classify ECs in 2 different phenotypes: persistent elite controllers (PCs), who maintain the virological control indefinitely over time; and transient elite controllers (TCs), who lose the virological control after being able to control viral replication without ART (5, 8).

Previous studies have focused on distinguishing both phenotypes, PC and TC, to determine the causes of the loss of the virological control and to define the best model of persistent viral remission. It is known that PCs have different immunological (5), proteomic (9), metabolomic (10), microRNA (11), and virological profiles (5) compared with TCs before losing the virological control. TCs, in comparison with PCs, are characterized by displaying a weaker HIV-specific T cell response with more limited polyfunctionality (5), a higher expression of markers associated with inflammation (9), a dysregulation of metabolic parameters (10, 11) and a higher viral diversity in *env* and *gag* genes (5) even one year before losing virological control.

Together, these results suggest that PCs can serve as a premier model for understanding immune pathways responsible for spontaneous cures of HIV and for designing new HIV cure and treatment strategies (12). Nevertheless, the mechanisms that allow PCs to maintain viral control in the absence of ART have not been fully characterized. Studies focused on this phenotype, also known as exceptional ECs (13), concluded that PCs generally present a low HIV reservoir and absence of viral diversity and evolution in HIV-1 *env* and *gag* sequences (5, 13).

Next-generation sequencing (NGS) techniques have changed the paradigm in the characterization of HIV reservoirs. NGS amplifies and sequences single, near full-length (NFL) HIV-1 proviruses, distinguishing between intact and defective provirus and identifying the integration site of the provirus in the genome (14, 15).

A distinct proviral reservoir landscape has been associated with the natural control of the HIV-1 infection (16–19), in which intact proviruses from a subset of ECs were preferentially integrated into centromeric satellite DNA or in Krüppel-associated box domain containing zinc finger genes, related to heterochromatin features (20) that do not support effective viral transcription (16). These findings suggest that the quality rather than the quantity of the HIV reservoir plays a crucial role in the search of a functional cure for HIV-1 infection (16). Nevertheless, the mechanisms that lead to this distinct, beneficial, intact proviral reservoir in ECs remain unclear, and may be associated with immune-mediated selection factors that preferentially eliminate intact proviruses in accessible chromatin (18, 21).

Despite all these advances, the HIV reservoir quality in PCs and TCs is unknown. It is important to elucidate the multiple mechanisms involved in the persistent natural virological control (22) since it would facilitate the future design of immunotherapies to bias the reservoir to a deep latency state compatible with permanent virological remission off ART. In this work, we comprehensively characterize the HIV reservoir and immunological footprints in TCs before losing the virological control compared with PCs who have been controlling the virus for a median of 25 years in absence of ART.

## Results

### Characteristics and clinical parameters of study participants.

**Figure 1:**
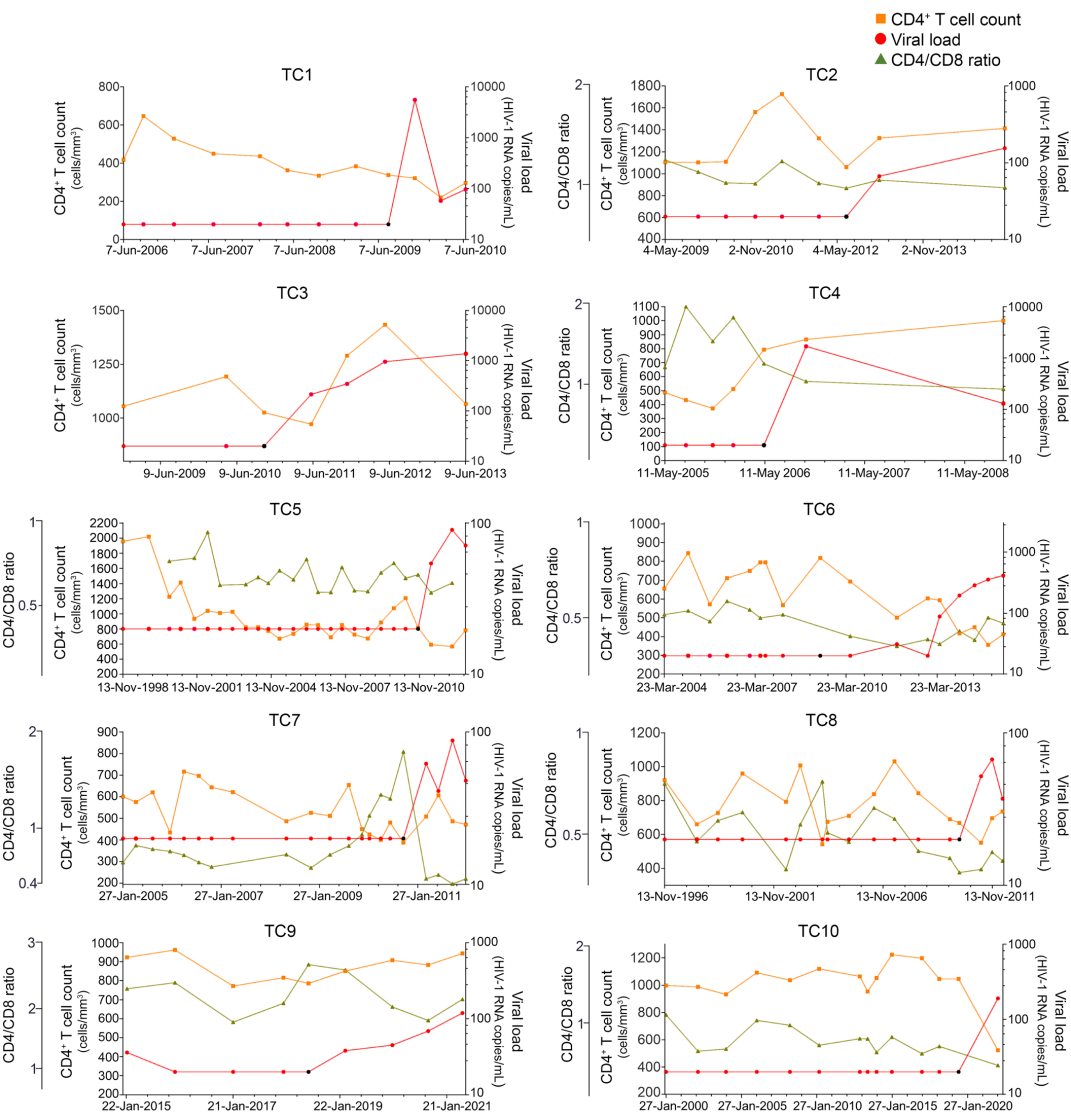
CD4^+^ T cell counts, viral load levels, and the CD4/CD8 ratio in TCs. CD4^+^ T cell counts and the CD4/CD8 ratio were represented in the left axes (CD4^+^ T cell count in orange and CD4/CD8 ratio in green) and viral load levels in the right axes (red). CD8^+^ T cell counts were unavailable in TC1 and TC3. The black dot represents the studied time point that preceded the loss of the virological control.

Twenty-seven ECs, with undetectable viral load in the absence of ART for at least 1 year of follow-up, were included in the study (5). Ten ECs were classified as TCs after experiencing a loss of virological control during more than 1 year of follow up, and 17 as PCs after maintaining persistent virological control during the follow-up period (5). Characteristics and clinical parameters of PCs and TCs are detailed in Table 1. The PC group was older and presented a longer time since HIV diagnosis than the TC group (Table 1). There were no differences in the remaining variables. Fifty-three percent of PCs presented protective HLA-alleles, HLA-B27/B57, versus 20 percent of TCs. Viral loads (HIV-RNA copies/mL), CD4^+^ T cells (cells/mm^3^), and the CD4/CD8 ratio from the 10 TCs, before and after losing the virological control, are shown in Figure 1. The studied time points of the TCs preceded the loss of the virological control from 0.3 to 2 years. PCs have maintained undetectable viral load with no ART for a median of 25.5 [IQR, 22.3–31.3] years with a median of 791 [IQR, 647–1013] CD4^+^ T cells/mm^3^.

### Distinct HIV-1 reservoir landscape in PCs compared to TCs before losing virological control.

The analysis of proviral sequences was performed using full length individual proviral sequencing (FLIP-Seq). The number of cells assayed, clades, and the total, intact and defective proviral sequences of the PCs and TCs are detailed in Supplemental Table 1 and 2; supplemental material available online with this article; https://doi.org/10.1172/JCI174215DS1, respectively.

**Figure 2:**
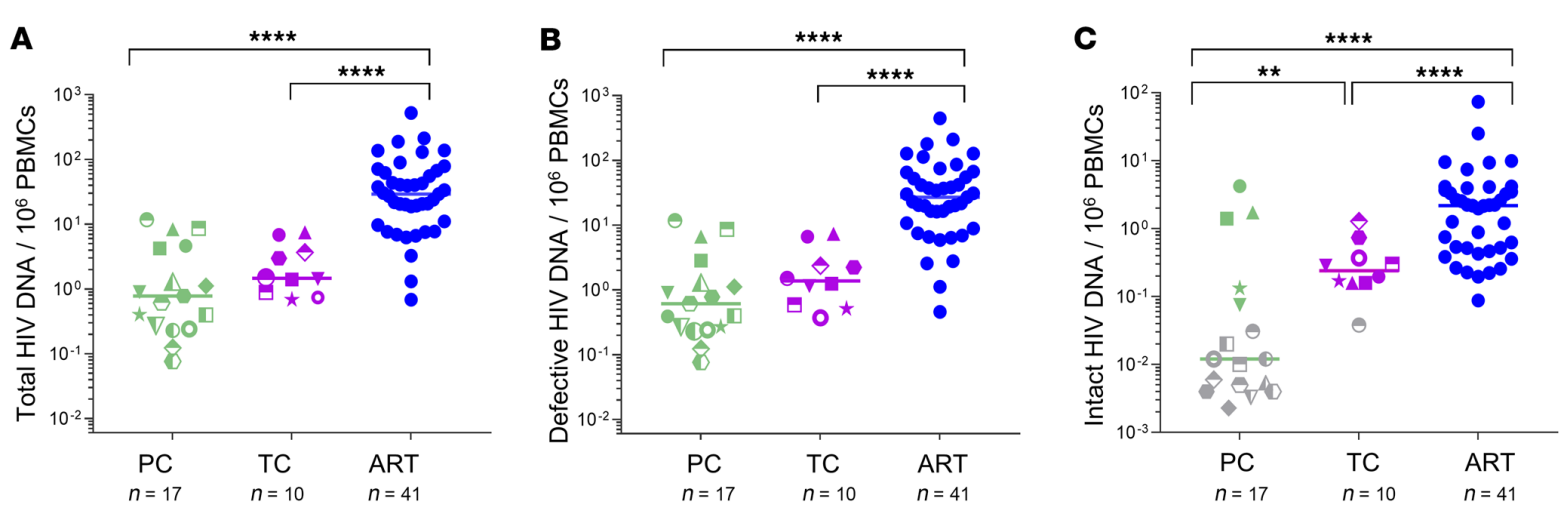
Analysis of HIV-1 proviral sequences in PCs, TCs, and participants on ART. (**A**) Total, (**B**) defective, and (**C**) intact provirus levels in PCs, TCs, and participants on ART. Grey dots represent values below the limit of detection (expressed as 0.05 copy/total number of analyzed cells without target identification). PCs and TCs are represented by unique identifiers (Supplemental Tables 1 and 2). Each dot represents a participant. Mann-Whitney *U* test was used to compare PCs, TCs, and participants on ART. *P* < 0.05 was considered statistically significant. **P ≤ 0.01, ****P ≤ 0.0001.

The individual HIV-1 proviral genome analysis was not different in the total (intact plus defective) (*P* = 0.167) (Figure 2A) nor the defective provirus levels (*P* = 0.141) (Figure 2B) between PCs (*n* = 17) and TCs (*n* = 10). Interestingly, the NFL intact provirus levels were significantly increased in TCs in comparison with PCs *(P* = 0.006) (Figure 2C) and importantly, no genome-intact HIV-1 was detected in 70.59% of PCs (indicated as grey dots in Figure 2C). Consequently, the intact/defective HIV-DNA ratio was significantly higher in TCs compared with PCs, counting the clones (*P* = 0.013) or not (*P* = 0.005) (Supplemental Figure 1, A and B, respectively). Significant differences were found in the total (Figure 2A), defective (Figure 2B), and intact provirus levels (Figure 2C) of PCs and TCs compared with participants on ART.

**Figure 3:**
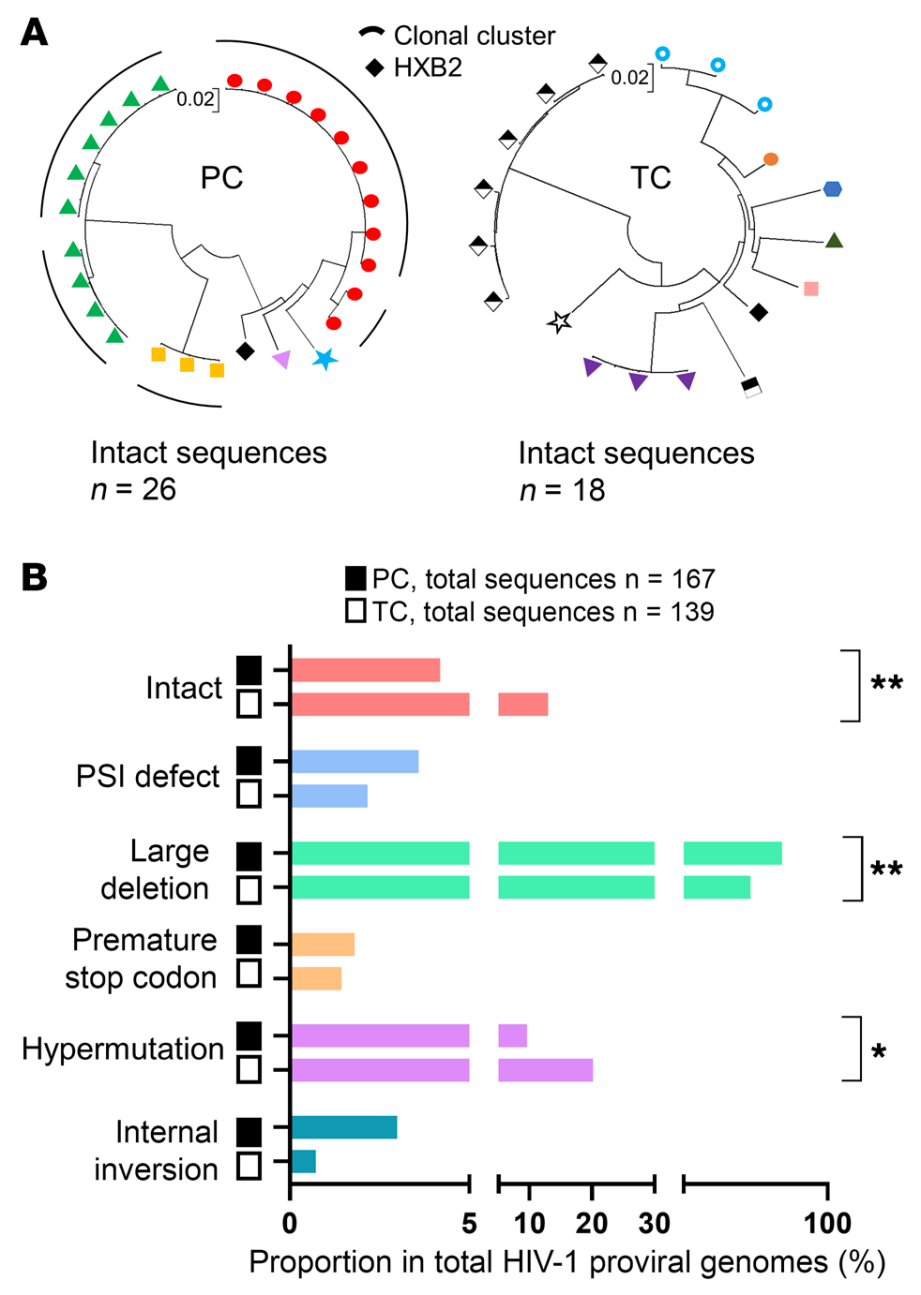
Genome-proviral sequences in PCs and TCs. (**A**) Circular maximum-likelihood phylogenetic trees for all genome-intact proviral sequences from PCs and TCs. HXB2 was used as the reference HIV-1 sequence. Dots with the same colors represent genome-intact proviral sequences from the same participant. Clonal sequences are indicated by black arches. PCs and TCs are represented by unique identifiers (Supplemental Tables 1 and 2). (**B**) Proportions of nonclonal genome-proviral sequences. Intact and defective proviruses as packaging signal defect (PSI), large deletion (LD), premature stop codon (PMSC) hypermutations and internal inversion, were included. FDR-adjusted 2-tailed Fisher’s exact tests were used to compare PCs and TCs. *P* < 0.05 was considered statistically significant. *P ≤ 0.05, **P ≤ 0.01.

PCs with higher levels of intact proviruses (*n* = 3) (Figure 2C) were derived from clonally expanded HIV-1 infected cells that accounted for 100% of all intact proviruses (Figure 3A; left panel). However, clonally expanded intact proviruses were not observed in TCs (Figure 3A; right panel). Further, significant differences were found in the proportion of nonclonal intact and defective sequences from PCs and TCs (Figure 3B). The proportion of intact proviruses (*P* = 0.006) and hypermutations (*P* = 0.013) were higher in TCs compared with PCs. However, the large deletion (LD) genome-defective provirus levels were lower in TCs compared with PCs (*P* = 0.004). No differences were found in the proportion of packaging signal defect (PSI) (*P* = 0.518), premature stop codon (PMSC) (*P* > 0.999) and internal inversion (*P* = 0.226) (Figure 3B) between PCs and TCs. Nevertheless, the proportion of sequences with internal inversion, counting clonal sequences, was significantly higher in PCs compared with TCs (*P* = 0.005), since internal inversion accounted for 57.9% of the HIV-1 total proviruses (81.8% were clonal) in the PC10 (Supplemental Figure 2).

### Distinct integration sites of intact genome proviruses in PCs compared with TCs before losing the virological control.

**Figure 4:**
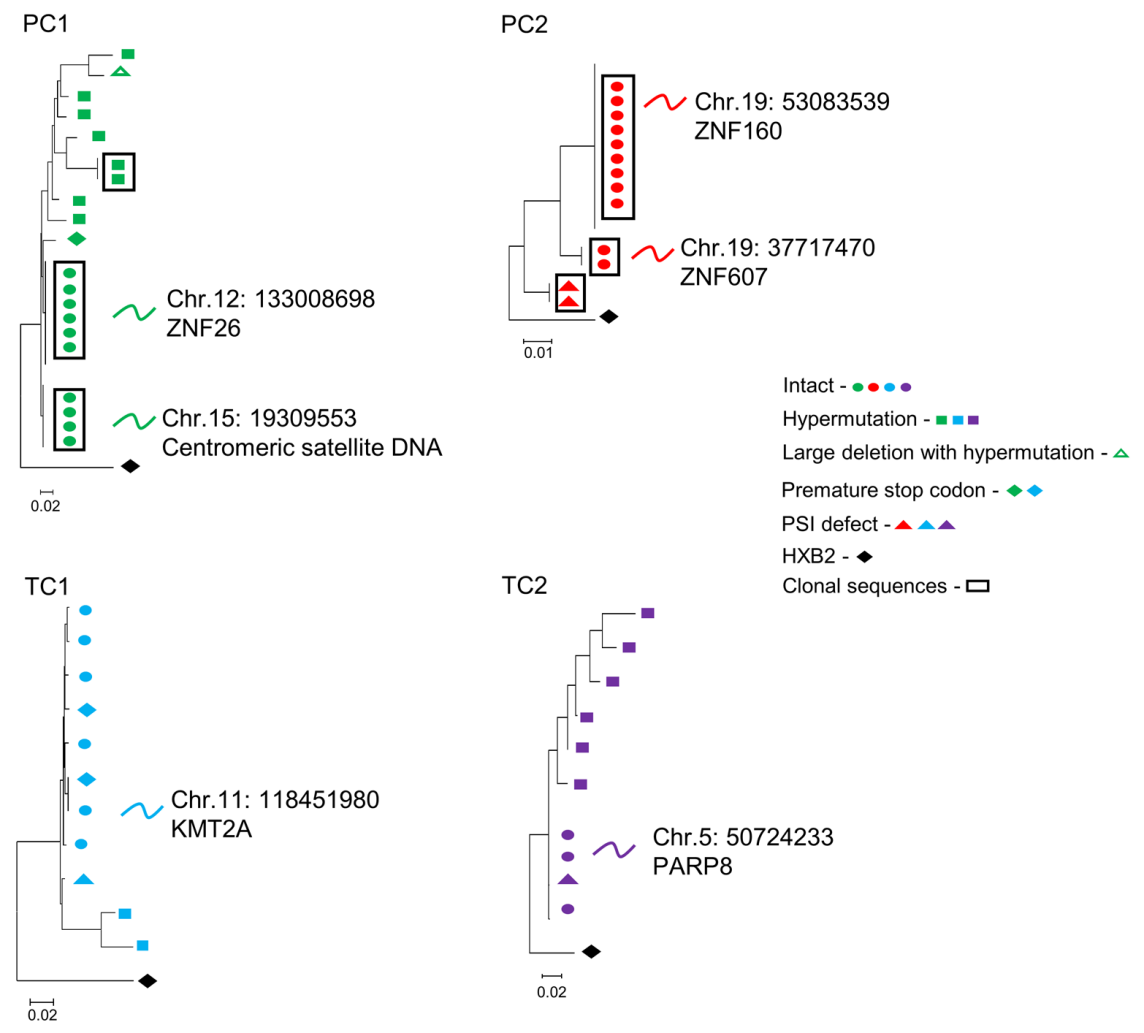
Simultaneous analysis of HIV-1 proviral sequences and integration sites in linear maximum-likelihood phylogenetic trees. Coordinates and relative positioning of integration sites are indicated. Clonal genome proviral sequences, defined by identical proviral sequences and identical corresponding integration sites, are highlighted in black boxes. The rest of the symbols represent different types of defective proviruses.

For a more in-depth analysis, together with FLIP-Seq, we assayed the corresponding intact provirus chromosomal integration site by matched integration site and proviral sequencing (MIP-Seq) and integration site loop amplification (ISLA). We analyzed the HIV-1 chromosomal integration sites of the intact proviruses of PCs and TCs (Figure 4). The intact provirus chromosomal integration sites are detailed in Supplemental Table 3. We focused on participants with intact genome proviruses and available samples to perform integration site analysis: 2 PCs (PC1 and PC2) that presented higher intact proviruses, derived from clonally identical sequences, and 2 TCs (TC1 and TC2) 0.3 and 0.6 years before losing the virological control, respectively. PC1 and PC2 have maintained ART-free HIV-1 control for 37 and 31 years, and TC1 and TC2 for 9 and 15 years until losing the virological control, respectively. PC1 presented 2 large clonal genome-intact proviral sequences located in centromeric satellite DNA and in genes that encode members of the Zinc Finger Nucleases (ZNF) protein family, ZNF26 (Figure 4; PC1). PC2 also presented 2 large clonal genome-intact proviral sequences, both located in ZNF genes, ZNF160 and ZNF607 (Figure 4; PC2) on chromosome 19. In contrast to PCs, TC1 and TC2 intact proviruses were located in genic regions before losing the virological control. Further, no clonal intact proviruses were detected in these participants (Figure 4; TC1 and TC2).

### Cell-associated HIV-1 RNA levels in PCs compared with TCs before losing virological control.

**Figure 5:**
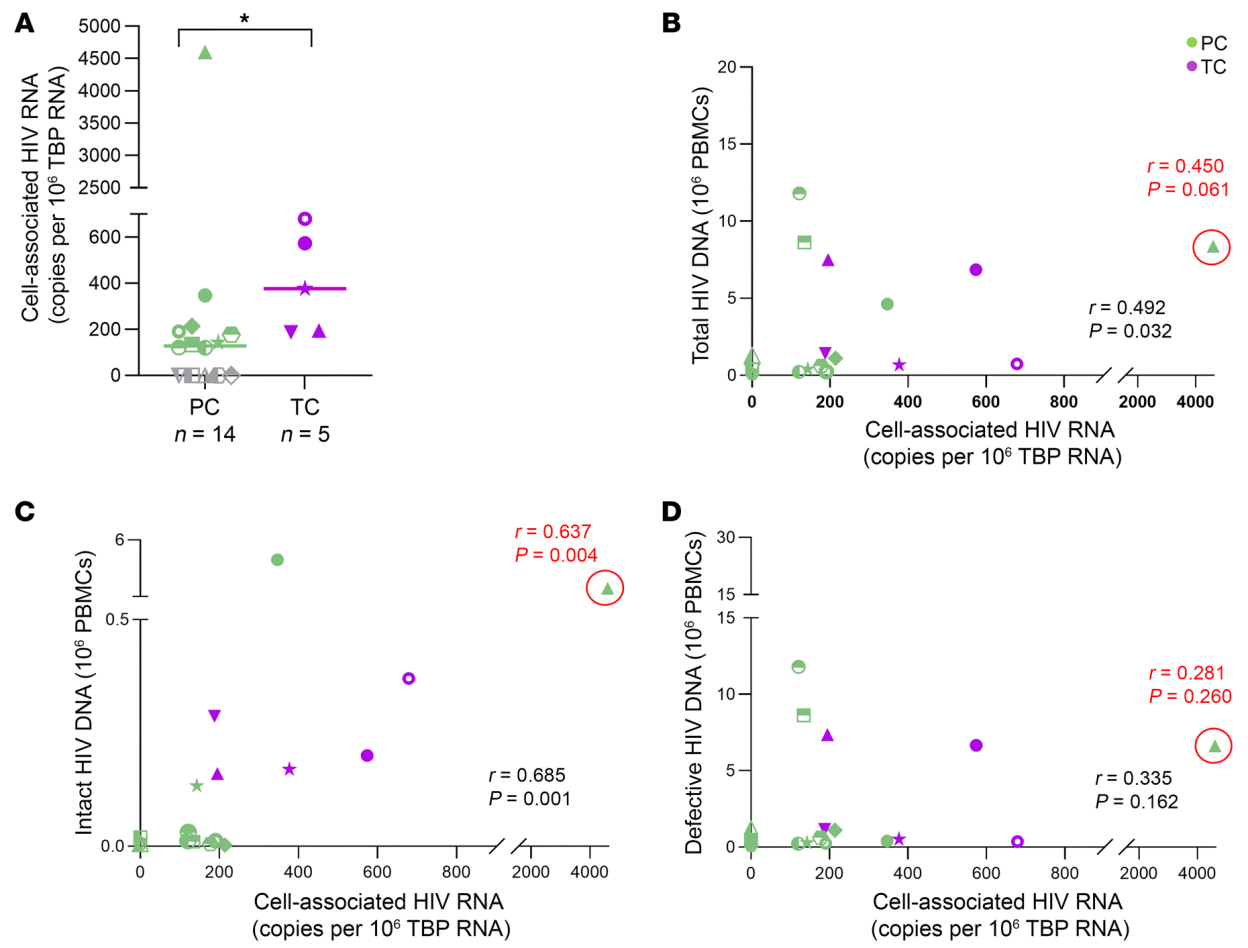
Cell-associated HIV-1-RNA in PCs and TCs. (**A**) Cell-associated HIV-1-RNA, expressed as copies per 10^6^ TBP RNA. Correlation between cell-associated HIV-1 RNA and (**B**) total, (**C**) intact and (**D**) defective genome proviruses in PCs and TCs. Each dot represents a participant. PCs and TCs are represented by unique identifiers (Supplemental Tables 1 and 2). Correlations were performed also excluding the participant in the red circle (PC1), in this case statistics are indicated in red. Mann-Whitney U test was used to compare PCs and TCs. *P* < 0.05 was considered statistically significant. *P ≤ 0.05. Spearman test was used for nonparametric correlations.

We also found differences in cell-associated HIV-1 RNA levels, which were higher in TCs (*n* = 5) compared with PCs (*n* = 14) (*P* = 0.018) (Figure 5A). Higher levels of cell-associated HIV-1 RNA detected in PCs corresponded to PC1 (4,602 copies per 10^6^ TATA-box binding protein (TBP) RNA) (Figure 5A and Figure 4; PC1) and PC2 (347 copies per 10^6^ TBP RNA) (Figure 5A and Figure 4; PC2). Interestingly, cell-associated HIV-1 RNA was not detected in 5 PCs who corresponded with those with no intact proviruses detected (grey dots in Figure 5A). Cell-associated HIV-1 RNA levels significantly correlated with total (Figure 5B) and intact proviruses (Figure 5C) but not with defective proviruses (Figure 5D). Excluding PC1, the correlation remained significant between cell-associated HIV-1 RNA and intact proviruses, finding a trend with total proviruses.

### Longitudinal analysis of viral reservoir landscape in PCs and TCs.

**Figure 6:**
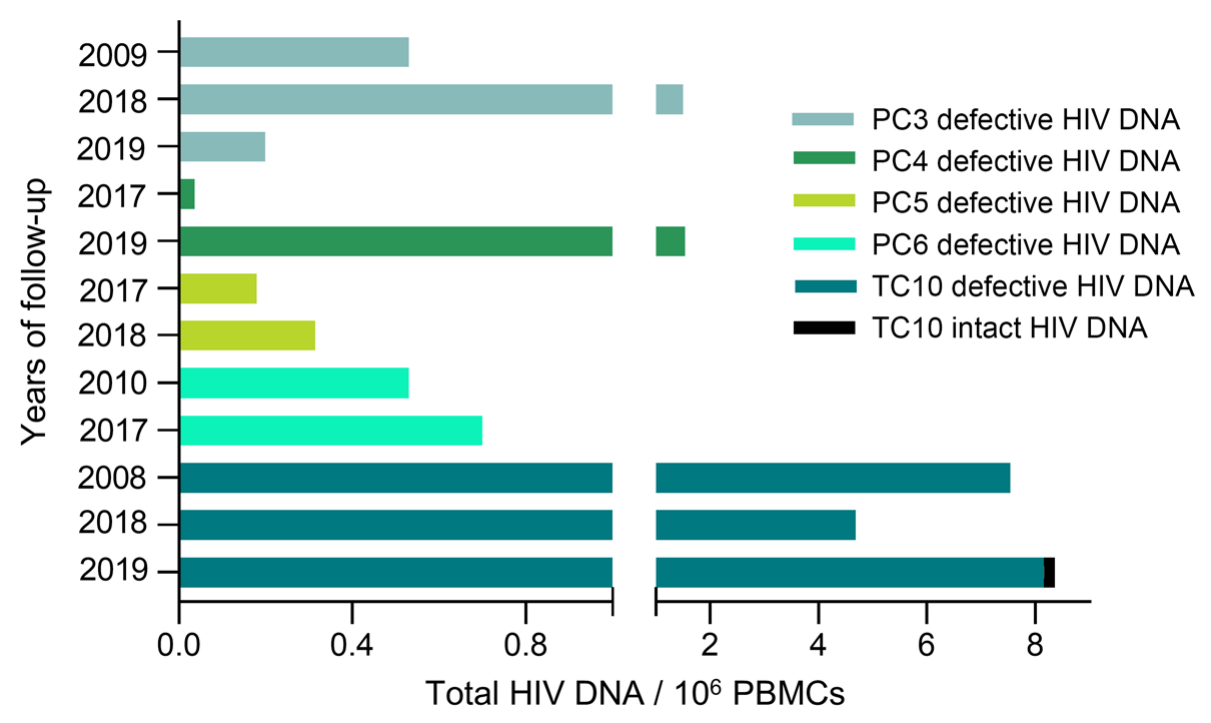
Longitudinal evolution of genome proviral reservoir landscape in PCs and TCs. Total, intact, and defective proviruses levels in PC3, 4, 5, 6, and TC10 over time.

Genome-intact HIV-1 proviruses were not detected in the 70.59% of the PCs (grey dots in Figure 2C). Consequently, we analyzed the reservoir of 4 of these PCs (PC3, PC4, PC5, and PC6) with available samples at different time points to evaluate possible changes in the viral reservoir over time (Figure 6). PC3 has maintained virological control for 27 years and no genome-intact HIV-1 proviruses were found in 3 different time points, 2009, 2018 and 2019, in a total of 21.6 million cells assayed. Differences in the defective provirus levels were found at different time points (Figure 6; PC3). PC4 has maintained virological control for 33 years and no genome-intact HIV-1 proviruses were found at 2 different time points, 2017 and 2019, in a total of 9.2 million cells. Differences in the defective provirus levels were found at different time points (Figure 6; PC4). PC5 has maintained virological control for 24 years and no genome-intact HIV-1 proviruses were found at 2 different time points, 2017 and 2018, in a total of 15 million cells. There were no differences in the defective provirus levels (Figure 6; PC5). PC6 has maintained virological control for 27 years and no genome-intact HIV-1 proviruses were found at 2 different time points, 2010 and 2017, in a total of 9.9 million cells. No differences were found in the defective provirus levels (Figure 6; PC6). We also analyzed the viral reservoir of one TC, TC10, who maintained virological control for 29 years, at 3 different time points, 13, 3, and 2 years before losing virological control, in a total of 8.2 million cells. Interestingly, intact HIV-1 proviruses were found 2 years before losing control, not being detected 13 and 3 years preceding aborted virological control (Figure 6; TC10). High defective provirus levels, mainly with hypermutations, were found even 13 years before losing virological control.

### Distinct signature of immune selection in intact and defective proviral sequences of PCs and TCs.

**Figure 7:**
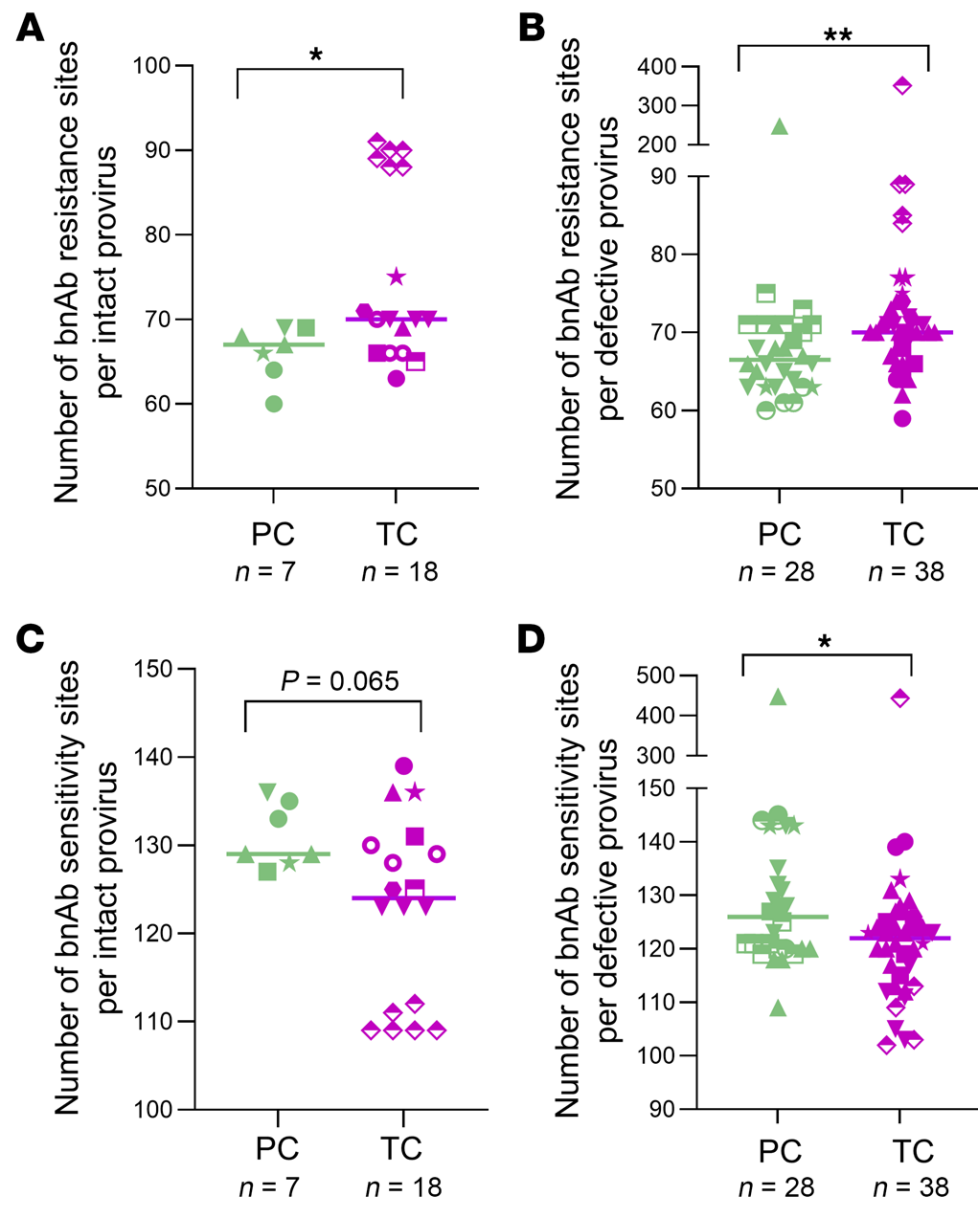
Analysis of bnAb resistance and sensitivity signature sites in intact and defective proviral sequences of PCs and TCs. Number of bnAb resistance sites per (**A**) intact and (**B**) defective provirus in PCs and TCs. Number of bnAb sensitivity sites per (**C**) intact and (**D**) defective provirus in PCs and TCs. Each dot represents an intact or defective proviral sequence. PCs and TCs are represented by unique identifiers (Supplemental Table 1 and 2). Mann-Whitney U test was used to compare PCs and TCs. *P* < 0.05 was considered statistically significant. *P ≤ 0.05, **P ≤ 0.01.

We analyzed the frequencies of amino acid variations associated with sensitivity or resistance to broadly neutralizing antibodies (bnAbs) recognizing the CD4 binding site, the V2/V3 envelope regions, or the membrane proximal external region (MPER), as previously reported (23), per intact and defective proviral sequence in PCs and TCs. Overall, TCs presented higher number of amino acid changes associated with resistance to bnAbs per intact (*P* = 0.021) (Figure 7A) and defective provirus (*P* = 0.007) (Figure 7B) than PCs. These differences were more remarkable for antibodies targeting the CD4 binding site (*P* = 0.011) and for antibodies recognizing the V2 envelope region (*P* < 0.0001) in intact provirus (Supplemental Figure 3A) and MPER regions (*P* = 0.080) in defective proviruses (Supplemental Figure 3B). Interestingly, intact-genome sequences with higher number of bnAb-resistance sites in TCs (Figure 7A) belonged to the participant who was closer to losing virological control, 0.3 years before the loss (Figure 4; TC1). On the contrary, PCs presented higher frequencies of amino acid variations associated with sensitivity to bnAbs per intact (*P* = 0.065) (Figure 7C) and defective proviruses (*P* = 0.020) (Figure 7D). These differences were more pronounced for antibodies recognizing the V2 envelope region (*P* = 0.023) and MPER region (*P* = 0.046) (Supplemental Figure 4A) in intact provirus and the CD4 binding sites (*P* = 0.047) and V3 envelope region (*P* = 0.021) in defective proviruses (Supplemental Figure 4B). Unexpectedly, the frequencies of amino acid variations associated with sensitivity to bnAbs recognizing the V3 envelope region in intact proviruses were significantly higher in TCs (*P* = 0.020) (Supplemental Figure 4A) whereas the frequencies of amino acid variations associated with resistance to bnAbs recognizing the same region were lower (*P* = 0.022) compared with PCs (Supplemental Figure 3A).

We next analyzed signs of cytotoxic T lymphocyte–driven (CTL-driven) immune pressure in the proviral sequences of PCs and TCs. No differences were found in the proportion of WT CTL or escape variant per intact (Supplemental Figure 5, A and B) and defective proviruses (Supplemental Figure 5, C and D) of PCs and TCs.

### Distinct Gag-specific T cell responses in PCs and TCs.

**Figure 8:**
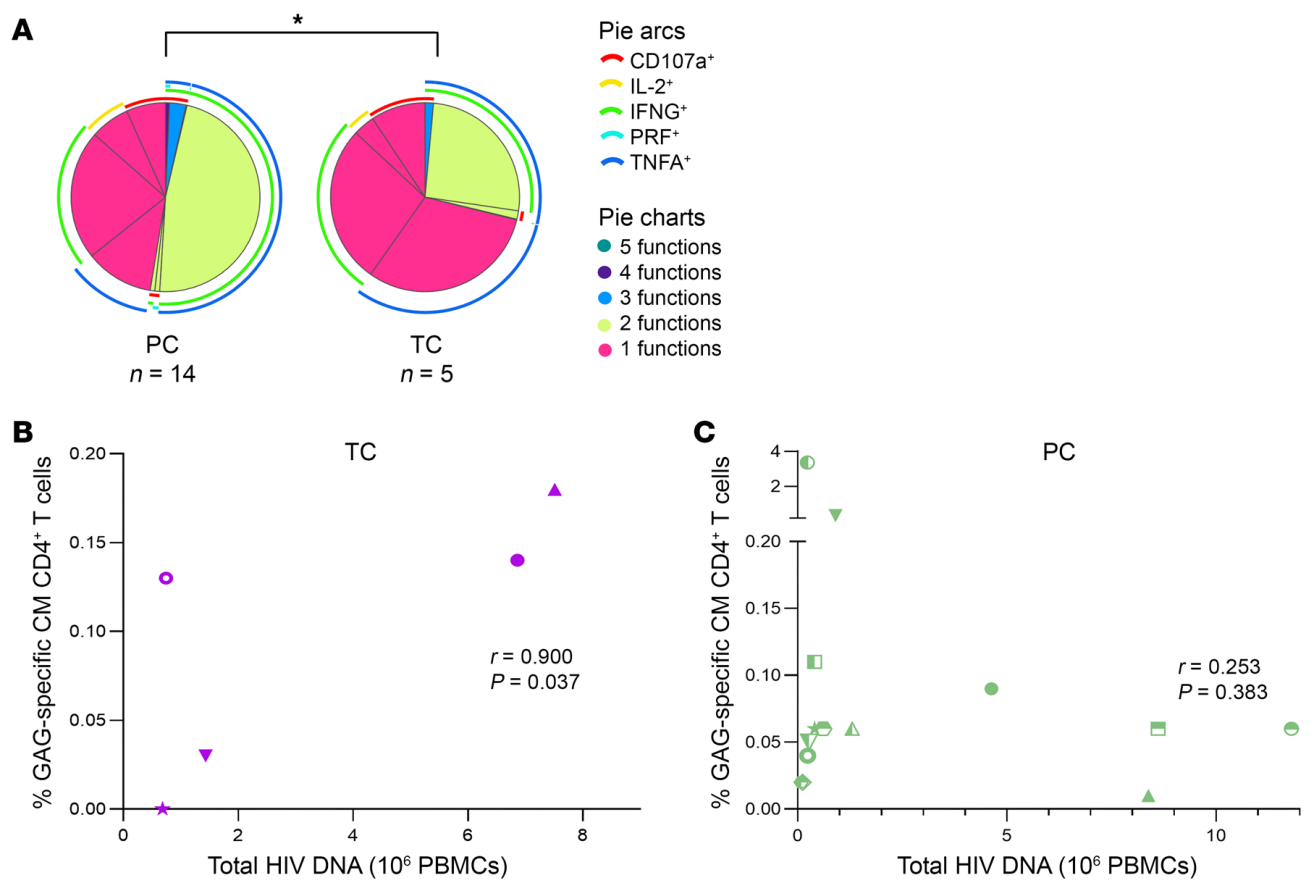
HIV-1-specific T-cell response in PCs and TCs. (**A**) HIV-1-specific CM CD4^+^ T cell polyfunctionality with up to 5 functional responses to Gag stimulation per T cell in PCs and TCs. The 5 functional responses to Gag stimulation represent the simultaneous production of IFNG, TNFA, IL-2, CD107a, and PRF per T cell. IFNG, TNFA, IL-2, CD107a, and PRF are shown in arcs in the polyfunctional distribution. Pestle and Spice were used for analysis. Correlations between Gag-specific CM T cell response with total HIV DNA levels (10^6^ peripheral blood mononuclear cells [PBMCs]) in (**B**) TCs and (**C**) PCs. Each dot represents a participant. PCs and TCs are represented by unique identifiers (Supplemental Table 1 and 2). Spearman test was used for nonparametric correlations. *P ≤ 0.05.

Immunological differences between PCs and TCs have previously been associated with the loss of the virological control (5). We compared and associated immune parameters of 14 PCs and 5 TCs with the quality of the HIV-1 reservoir. No differences in the magnitude of the response, assayed by cytokine production (IL-2, TNFA, and IFNG) after stimulation with Gag peptides in PCs and TCs, were observed neither in CD4^+^ nor in CD8^+^T cells (for gating strategy, see Supplemental Figure 6). However, a lower frequency of polyfunctionality, defined as simultaneous production of IFNG, TNFA, IL-2, CD107a, and perforin (PRF) per T cell in response to Gag stimulation, was observed in central memory (CM) CD4^+^ T cells in TCs compared with PCs (*P* = 0.049) (Figure 8A). Significant positive correlations were found between total proviruses and the frequency of HIV-specific total memory (*P* = 0.037; r=0.900) and CM CD4^+^ T cell response (*P* = 0.037; r=0.900) (Figure 8B) in TCs but not in PCs (*P* = 0.311; r=0.292 and *P* = 0.383; r=0.253, respectively) (Figure 8C).

### Distinct HIV-specific CD8^+^ T cell proliferation in PCs and TCs.

**Figure 9:**
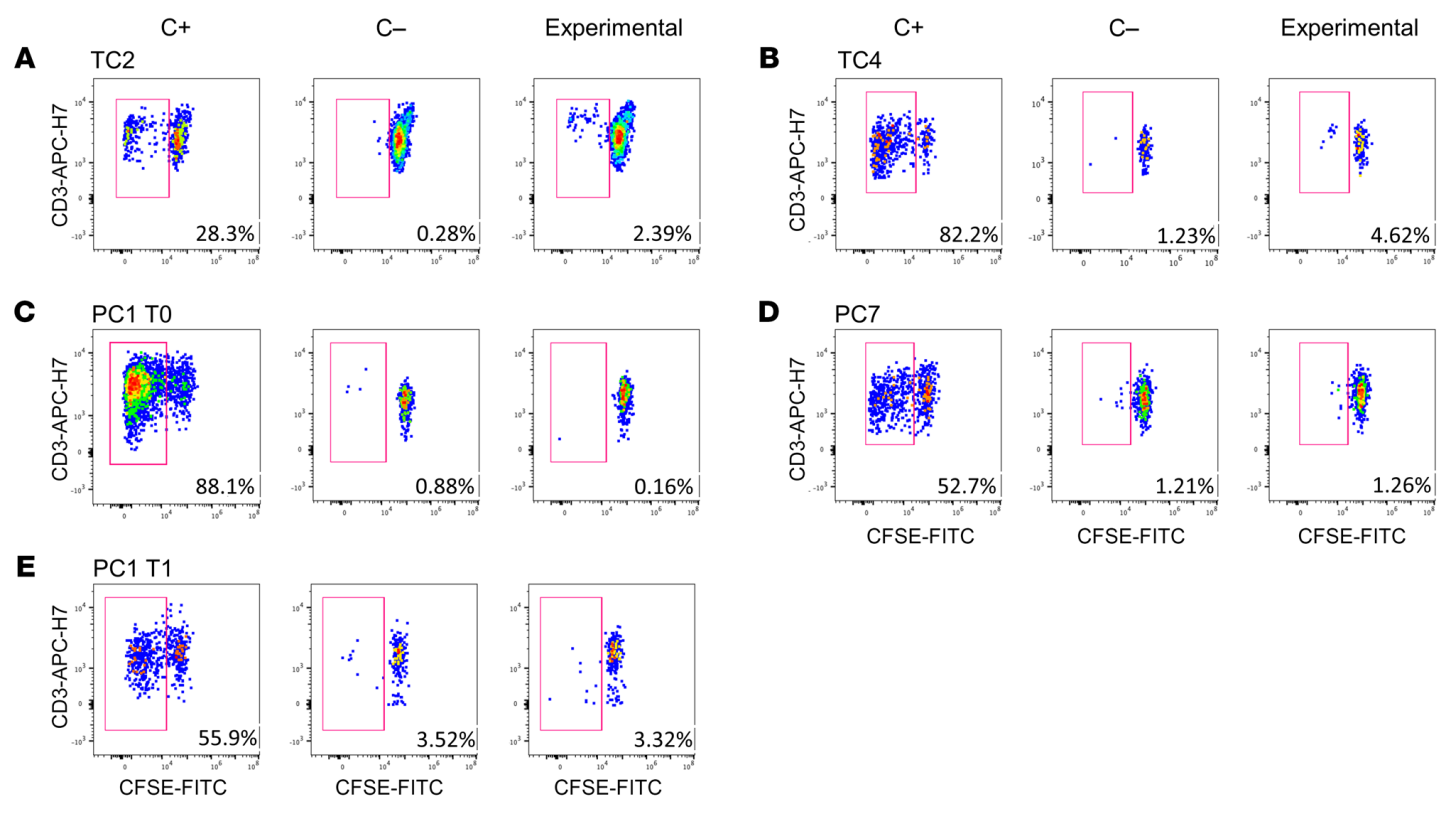
HIV-specific CD8^+^ T cell proliferation assay. (**A**) TC2, (**B**) TC4, (**C**) PC1 T0, (**D**) PC7, and (**E**) PC1 T1 (1 year after T0). C+: stimulated PBMCs with Staphylococcal enterotoxin B (SEB) (left panel). C–: unstimulated PBMCs (middle panel). Experimental: stimulated PBMCs with HIV (Gag)-specific peptide (right panel) after 5 days in culture.

Given the importance of the CD8^+^ T cell role in the spontaneous virological control (24), we analyzed CD8^+^ T cell proliferation in PCs and TCs after stimulation with an HIV (Gag)-specific peptide pool. Comparing the experimental condition (Figure 9, A–D; right panel) with the negative control (Figure 9, A–D; middle panel) we found that TC2 (Figure 4; TC2) and TC4 (Figure 9, A and B, respectively) presented a higher CD8^+^ T cell proliferation before losing the virological control than PC1 (Figure 4; PC1) and PC7 (Figure 9, C and D, respectively).

After that, to prove the lack of proliferation in PCs, we analyzed the CD8^+^ T cell proliferation in 2 longitudinal samples, T0 (Figure 9C) and T1 (Figure 9E), 1 year after T0, of PC1 (Figure 4; PC1). The CD8^+^ T cell proliferation was not changed over time, since no differences were found between experimental (Figure 9, C and E; right panel) and negative control (Figure 9, C and E; middle panel) in any of the time points. The positive control stimulated with *Staphylococcal enterotoxin B* (SEB) validated the efficacy of the assay (Figure 9, A–E; left panel).

### Thymic function in PCs compared with TCs before losing virological control.

**Figure 10:**
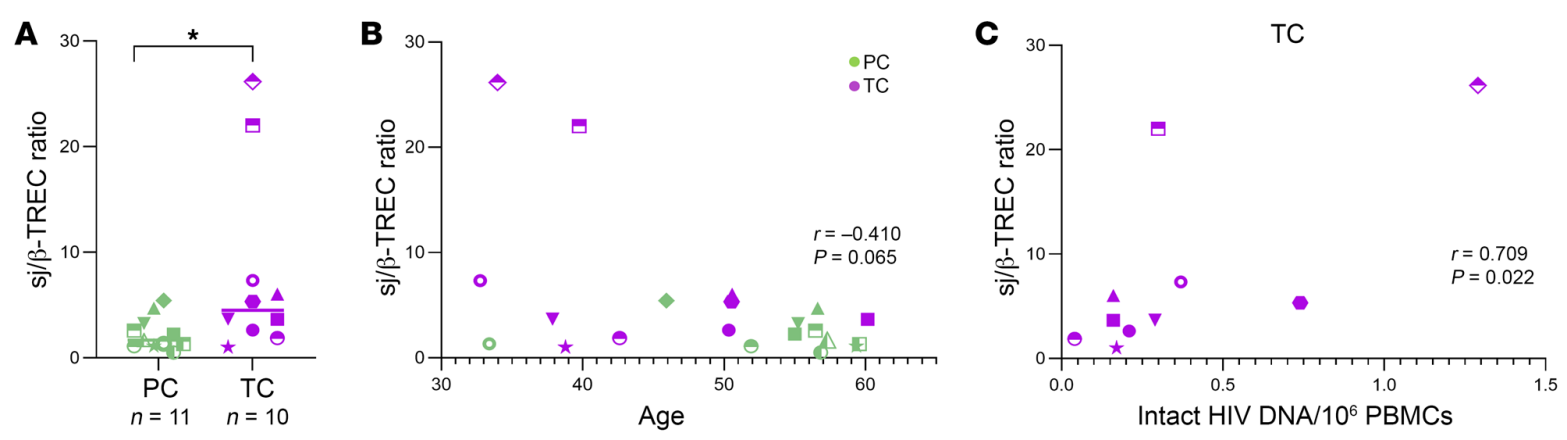
Thymic function in PCs and TCs. (**A**) Dot graphs represent sj/β-TREC ratio. (**B**) Correlations between sj/β-TREC ratio and age in PCs sand TCs. (**C**) Correlations between sj/β-TREC ratio and the frequency of intact HIV DNA (10^6^ PBMCs) in TC. Each dot represents a participant. PCs and TCs are represented by unique identifiers (Supplemental Table 1 and 2). Mann-Whitney U test was used to compare PCs and TCs. Spearman test was used for nonparametric correlations. *P* < 0.05 was considered statistically significant. *P ≤ 0.05.

Thymic function has been associated with HIV disease progression (25). We assayed the sj/β-TREC ratio in 11 PCs and 10 TCs by droplet digital PCR (ddPCR). We observed that sj/β-TREC ratio was significantly increased in TCs in comparison with PCs (*P* = 0.024) (Figure 10A). Nevertheless, these differences were colineal with age, as we observed an inverse correlation between age and sj/β-TREC ratio (r = –0.410; *P* = 0.065) (Figure 10B) and TCs were younger than PCs (Table 1). However, a significant positive correlation was found between sj/β-TREC ratio and the levels of intact proviruses in TCs (r = 0.709; *P* = 0.022) (Figure 10C) but not in PCs (data not shown).

## Discussion

Despite the recent advances in measuring and characterizing the HIV reservoir (26, 27), the characteristics and distinctive features of PC and TC HIV-1 reservoirs remain poorly defined. Understanding the mechanisms responsible for the maintenance and loss of virological control in ECs is important to design functional or sterilizing cure strategies.

The present study, which comprehensively analyzed the quality of the TC HIV-1 reservoir before losing virological control, demonstrates a distinct and dynamic viral reservoir landscape in TCs compared with PCs. Our analysis showed, as indicative factors of the viral rebound, higher intact proviruses and cell-associated HIV-1 RNA levels, and a higher viral diversity with no detectable intact provirus clones in TCs compared with PCs. Importantly, intact proviruses may be more likely to be located in permissive genic euchromatic positions in TCs, although our results are limited by sample size. These findings are crucial since they could be consistent with TCs having subclinical HIV-1 replication before losing virologic control; analysis of additional measures of active virus replication are suggested to test this hypothesis further. Despite that no differences were observed in defective provirus levels between PCs and TCs, a higher proportion of hypermutations was found in TCs and may facilitate the HIV-1 escape from the immune system by increasing the genetic diversity and the evolution of viral variants, as previously reported (28). As opposed to TCs, genome-intact proviral sequences were not detected in 70.59% of PCs after analyzing millions of cells. It is notable that no cell-associated HIV-1 RNA was detected in 5 of these PCs. The absence of genome-intact proviral sequences in a large number of analyzed cells has previously been associated with a spontaneous cure of HIV-1 infection in the Berlin (16, 29) and Esperanza patients by the same techniques used in this study (30). Although we cannot confirm with our data that these participants, with virological control without ART for a median of 25 years, have achieved a spontaneous cure of HIV-1 infection, further studies can now be guided by our findings to study a larger number of cells from PBMCs and different anatomical compartments (31), such as lymphoid tissue, known for containing the majority of the viral reservoir (32, 33). These results suggest that the idea of a possible spontaneous cure of HIV-1 in the Esperanza patient is not anecdotic, and a higher number of ECs with this reservoir profile might be spontaneously cured. This hypothesis is supported by the longitudinal analysis of the HIV-1 reservoir in 4 of the PCs. Genome-intact HIV-1 proviruses were not detected in these participants during the follow up, some of them with samples 10 years apart. The detectable proviral DNA was completely defective in all the studied time points and, thus, unable to produce infectious virions. These data strongly suggest the absence of enough genome-intact proviruses to cause loss of control and support additional research to strengthen evidence for this hypothesis

The other profile found in PCs consisted of participants who presented higher genome-intact proviral levels, derived completely from clonally expanded HIV-1 infected T cells and preferentially located in centromeric satellite DNA or ZNF genes, both associated with heterochromatin regions, generally disfavored for proviral integration and linked to deep viral latency (20, 34–36). Moreover, clonal-intact proviral sequences presented the same integration site, confirming their role in the reservoir persistence in PCs (37). Interestingly, both large clones in PC2 were integrated in ZNF genes (37) located in defined regions that are occupied by heterochromatin proteins of chromosome 19 (20, 34), as previously reported in a subset of ECs (16). Unlike PCs, we did not detect intact clonally expanded HIV-1 infected cells in TCs (5). These data suggest that the intact proviral reservoir of PCs, in contrast with TCs, seems mostly fueled by clonal proliferation of latently infected cells harboring early seeded intact proviruses. This fact resembled our previous findings in the overall EC population (16), probably because the more comprehensive analysis was performed in those ECs with intact proviral clones, consistent with the PC phenotype described in the present study.

In addition, cell-associated HIV-1 RNA levels were significantly higher in TCs and positively correlated with total and intact proviruses. Notably, higher cell-associated HIV-1 RNA levels in PCs corresponded to PC1 and PC2, the participants that presented clonal intact provirus sequences located in centromeric satellite DNA or ZNF genes, reassuring the importance of the quality, rather than the quantity, of viral reservoir. This fact may indicate a production of viral proteins by defective proviruses in PCs (38), mostly driven by the higher proportion of LD proviruses observed in this phenotype (39) that could act as a therapeutic vaccine and could magnify the antiviral host immune activity in PCs.

Regarding the immune pressures that preceded the viral rebound, we found a lower number of bnAb sensitivity sites and, conversely, a higher number of bnAb resistance sites per intact and defective proviruses in TCs compared with PCs. These findings may be consistent with more selection among TC with virus resistance to humoral immune responses, but our data are not yet definitive as to whether this could result from the observed higher viral diversity in TCs or if selection for resistance was also occurring. Curiously, we found the opposite phenomenon for bnAbs that recognize the V3 envelope region in intact genome proviruses, lower sensitivity, and higher resistance in PCs, compared with TCs, suggesting that the V3 envelope region may not be as important to viral control as the CD4 binding site, V2, and MPER regions. Interestingly, PC1, the participant that presented higher bnAb-resistance sites per defective provirus in PCs, was the same participant that presented clonal intact provirus sequences located in centromeric satellite DNA and ZNF genes and higher cell-associated HIV-1 RNA levels. This finding confirms the persistence of defective provirus and its role in the production of viral proteins (38), and, consequently, in the antiviral immune response in PCs, as mentioned previously. However, the absence of differences in WT and CTL escape mutations may be biased by the fact that only clade B consensus sequences were analyzed, since clade A1 and F1 HIV-infected participants, including PC1 and TC1, were not included in the analysis due to a lack of information related to escape mutations for these clades.

All these immunological proviral footprint data are in accordance with the lower T cell polyfunctionality found in TCs compared with PCs, as previously reported (5). Interestingly the reduced quality of the Gag-specific T cell response in TCs was intimately associated with viral reservoir measurements. In effect, HIV-specific CD4^+^ T cells are known to be infected by the virus at higher frequencies than other memory CD4^+^ T cells (40). Moreover, active reservoirs have previously been reported to be enriched in CM T cells (41). These facts may explain the positive correlation found between HIV-specific CM response in CD4^+^ T cells with total proviruses in TCs, but not in PCs, again pointing out the ongoing HIV infection of these HIV-specific CD4 memory T cells in TCs.

These data were also associated with thymic function levels, which have previously been associated with HIV disease progression (25), in fact, the higher levels of thymic function in TCs compared with PCs positively correlated with intact-genome proviruses. Interestingly, the highest sj/β-TREC ratio belonged to TC1, the participant closer to lose the virological control. These data may indicate a compensatory mechanism of the adaptive immune system to maintain T cell number and suppress ongoing viral replication, resulting in the loss of the virological control due to the decreased T cell polyfunctionality.

A main limitation of our study was the sample availability and consequently the need for more immunological data, especially in TCs, and above all, the number of sequences available per participant, particularly in PCs with no intact proviral sequences detected. We partially counteracted this limitation with the longitudinal analysis performed in participants with this profile. Additionally, it is notable that these participants are exceptional, and analyzing a larger number of cells does not guarantee finding more sequences, as it has been shown in participants with unique reservoir profiles such as the Esperanza patient, HIV pediatric patients, and a subgroup of ECs (18, 30, 42).

Together, we observed absence of detectable intact provirus sequences and a deep viral latency in PCs, which seems to follow a “block and lock” mechanism (43), by silencing of intact proviral gene expression through chromosomal integration into repressive chromatin locations. By contrast, higher intact-genome proviral levels, transcriptionally active and with higher resistance to immune recognition, were observed in TCs before losing the virological control. Despite the higher thymic function and CD8 T cell proliferation found in TCs, their lower Gag-specific T cell polyfunctionality may contribute to the loss of the virological control.

In summary, our results showed a markedly distinct intact proviral reservoir and immunological landscapes associated with the loss and maintenance of persistent spontaneous HIV control. These findings are important, albeit not definitive, as they go one step further to identify the PC phenotype as the premier model of a functional cure, determine the causes of the loss of spontaneous viral control, and identify PCs and other PLHIV with this distinct reservoir signature as spontaneously cured. Our results emphasize that confirmation of these hypotheses to predict a higher likelihood of persistent spontaneous control will require a larger sample of longitudinally sampled controllers in ongoing cohorts.

## Methods

### Sex as a biological variable.

Cisgender women and men were included in the study.

### Study design and participants.

Participants were defined as ECs when viral load determinations were under the detection limit in the absence of ART for at least 1 year of follow up (5). Human PBMCs were collected from 27 ECs and 41 participants on ART. Ten ECs were classified as TCs and 17 as PCs. Participants were classified as TCs after experiencing a loss of virological control, sustained viral load above the detection limit during more than 1 year of follow up (at least 2 consecutive detectable viral loads), as previously reported (5). Using this classification, we have previously observed differences in immunological (5), proteomic (9), metabolomic (10), microRNA (11) and virological profiles (5) in PCs compared with TCs before losing virological control. Participants were classified as PCs after maintaining persistent virological control during the follow-up period.

TCs were selected based on sample availability less than 2 years before losing virological control, according to our previous findings (5). Frozen PBMCs of these participants were obtained from Spanish HIV Hospital Gregorio Marañón (HGM) BioBank belonging to the AIDS Research Network (44) and collecting participants’ clinical data from Red de Investigación en Sida (RIS) Controllers Study Group Cohort (ECRIS) (45). PC samples were collected from Virgen del Rocio and Virgen Macarena University Hospitals, Seville, Spain; Lausanne University Hospital, Lausanne, Switzerland; Virgen de las Nieves University Hospital, Granada, Spain; Reina Sofía University Hospital, Córdoba, Spain; Costa del Sol and Virgen de la Victoria Hospital, Málaga, Spain; Torrecardenas University Hospital, Almeria, Spain, and Joan XXIII University Hospital, Tarragona, Spain. Samples of participants on ART were collected from Massachusetts General Hospital (MGH). Viral load and frozen PBMCs from PCs were obtained from healthcare providers.

### PBMC isolation.

PBMCs were isolated using BD Vacutainer CPT Mononuclear Cell Preparation Tubes (BD Biosciences), with sodium heparin as anticoagulant, by density gradient centrifugation at the same day of blood collection. CPTs were centrifuged at 1,811*g* for 20 minutes at room temperature (RT). Afterward, PBMCs were cryopreserved in freezing medium (90% FBS [Thermo Fisher Scientific] and 10% DMSO [PanReac AppliChem]) in liquid nitrogen until further use.

### DNA and RNA extraction.

Genomic DNA and RNA were extracted from PBMCs using a blood DNA minikit (Omega Bio-Tek) and NucleoSpin RNA purification kit (Macherey-Nagel), respectively. DNA and RNA were quantified using the Qubit assay (Thermo Fisher Scientific) according to the manufacturer’s instructions.

### HIV-1 DNA quantitation.

HIV-1 DNA was quantified from previously extracted DNA by ddPCR using the BIO-RAD QX200 Droplet Reader, as previously reported (46). The PCR program was run according to the manufacturer’s protocol using an annealing temperature of 58°C. Primers and probes targeting gag regions and the viral 5′ long terminal repeat (LTR) were: 6F (5′-CATGTTTTCAGCATTATCAGAAGGA-3′), 84R (5′-TGCTTGATGTCCCCCCA CT-3′) and probe (5′-VIC-CCACCCCACAA GATTTAAACACCATGCTAA-BHQ1-3′); LT forward (5′-TGTGTGCCCGTCTGTTGTGT-3′), LT reverse (5′-GCCGA GRCCTG CGTCGAGAG-3′) and the LT probe (5′-FAM (6-carboxyfluorescein)-CAGTGGCGCCCGAACAGGGA-BHQ1-3′). Ribonuclease P protein subunit p30 (RPP30) was used as a housekeeping gene to normalize HIV-1 DNA copies. The following primers and probes were used to quantify RPP30: RPP30 forward (5′-GATTTGGACCTGCGAGCG-3′), RPP30 reverse (5′-GCGGCTGTCTCCACA AGT-3′), and probe (5′-VIC-CTGACCTGAAGGCTCT-BHQ1-3′). Data were analyzed using Bio-Rad QuantaSoft software version 1.7.4.

### Cell-associated HIV-1 RNA quantitation.

Cell-associated HIV-1 RNA was quantified from previously extracted RNA by ddPCR with the One-Step RT-ddPCR kit (Bio-Rad) using the BIO-RAD QX200 Droplet Reader, as previously reported (47). The PCR program was run according to the manufacturer’s protocol using an annealing temperature of 58°C and the same primers and probes as previously described (see section HIV-1 DNA quantitation). TBP was used as a housekeeping gene to normalize HIV-1 RNA copies. The following primers and probes were used to quantify TBP: TBP forward (5′-CACGAACCACGGCACTGATT-3′), TBP reverse (5′-TTTTCTTGCTGCCAGTCTGGAC-3′), and probe (5′-HEX-TGTGCACAGGAGCCAAGAGTGAAGA/3-IABkFQ-3). Data were analyzed using the Bio-Rad QuantaSoft software version 1.7.4.

### FLIP-Seq.

Genomic DNA, previously extracted from PBMCs, was diluted to single proviral genomes based on ddPCR results (see section HIV-1 DNA quantification) and Poisson distribution statistics (1 provirus was present in approximately 20%–30% of wells). Afterward, DNA was subjected to HIV-1 near-full–genome amplification using a single-amplicon nested PCR approach, as previously reported (48). The following primers were used for the first and second-round nested-PCR, respectively: U5-623F (5′-AAATCTCTAGCAGTGGCGCCCGAAC AG-3′) and U5-601R (5′-TGAGGGATCTCTAGTTACCAGAGTC-3′); U5-638F (5′-GCGCCCGAACAGGGACYTGAAARCGAAAG-3′) and U5-547R (5′-GCACTC AAGGCAAGCTTTATTGAGGCTTA-3′). PCR products were visualized by 0.7 % agarose gel electrophoresis (Quantify One and ChemiDoc MP Image Lab; BioRad) and all NFL HIV-1 (≈ 8000 bp) were subjected to Illumina MiSeq sequencing at the MGH DNA Core facility.

Short reads were de novo assembled using Ultracycler version 1.0 and aligned to HXB2 to identify large deleterious deletions (< 8000 bp of the amplicon), out-of-frame indels, premature/lethal stop codons, internal inversions, or packaging signal deletions (≥ 15 bp insertions and/or deletions relative to HXB2) through an automated pipeline written in Python programming language (49). The presence/ absence of APOBEC-3G/3F–associated hypermutations was determined using Los Alamos National Laboratory (LANL) HIV-1 Sequence Database Hypermut 2.0 program (50). Viral sequences without any of the mutations previously mentioned were classified as intact genome sequences. An alternative analysis was used to classify a sequence as intact, as previously reported (51). Phylogenetic distances between sequences were determined through maximum-likelihood trees in MEGA and visualized with Highlighter plots. Clonality was determined by identical sequences, with 3 or fewer mismatches between proviral sequences, and integration sites. Nucleotide variations due to primer binding sites were not considered for clonality analysis.

### Integration site analysis.

MIP-Seq was used to profile the chromosomal locations of intact proviruses. Firstly, a whole genome amplification (WGA) was performed by a multiple displacement amplification (MDA) with Φ29 polymerase (REPLI-g Single Cell Kit) (Qiagen) (52), according to the manufacturer’s protocol. Subsequently, DNA from each well was split and separately subjected to viral sequencing and integration site analysis (48).

Integration sites of each intact provirus, obtained from the WGA, were identified by ISLA technique, as previously described (53). A second WGA was performed when it was necessary to increase the amount of DNA. PCR products were subjected to next-generation sequencing using Illumina MiSeq.

MiSeq paired-end FASTQ files were demultiplexed; small reads (142 bp) were then aligned simultaneously to human reference genome GRCh38 and HIV-1 reference genome HXB2 using bwa-mem (54). Biocomputational identification of integration sites was performed according to previously described procedures (53, 55). Briefly, chimeric reads containing both human and HIV-1 sequences were evaluated for mapping quality based on: (a) HIV-1 coordinates mapping to the terminal nucleotides of the viral genome, (b) absolute counts of chimeric reads, and (c) depth of sequencing coverage in the host genome adjacent to the viral integration site. The final list of integration sites and its corresponding chromosomal annotations was obtained using Ensembl, the UCSC Genome Browser and GENCODE. Repetitive genomic sequences harboring HIV-1 integration sites were identified using RepeatMasker.

### Sequence analysis.

Clades of intact HIV-1 proviral sequences were determined using the LANL HIV Sequence Database Recombinant Identification Program. For each clade B proviral sequence, optimal CTL epitope sequences restricted by autologous HLA class I alleles within 9 HIV-1 genes were identified (56), by best-defined HIV-1 CTL/CD8^+^ T cell epitopes from the LANL HIV Immunology Database. The CTL/CD8^+^ Epitope Variants and Escape Mutations were used from the same database to classify epitope sequences from each provirus as WT, escaped, or uncharacterized, according to the respective HIV-1 subtype and HLA allele. Thanks to an existing compendium of proviral sequence signature mutations that influence susceptibility to bnAbs (23), we evaluated the frequencies of amino acid variations associated with sensitivity or resistance to bnAbs. The sensitivity of proviral species to bnAbs was estimated by calculating the number of amino acid signature sites associated with sensitivity to 4 different bnAbs classes, which recognize the CD4 binding site, the V2/V3 envelope regions, or the membrane proximal external region (MPER), within the *env* amino acid sequence from each provirus, as previously described (23)

### CD8^+^ T cell proliferation assay.

PBMCs, 1 × 10^6^ cells/mL, were stained at 37°C for 20 minutes with 0.5 μM CellTrace carboxyfluorescein succinimidyl ester (CFSE; Thermo Fisher Scientific) according to the manufacturer’s protocol. Cells were washed twice with RPMI-1640 medium (RPMI) supplemented with 10% FBS (Sigma-Aldrich), and plated in 96-well round-bottom polystyrene plates, 200 μL per well. Experimental samples were incubated with 20 ng/mL of an overlapped HIV (Gag)-specific peptide pool (NIH AIDS Reagent Program). Positive control well was stimulated with 2 μg/mL of SEB (Sigma-Aldrich) and the negative control contained unstimulated PBMCs. Subsequently, cells were cultured for 5 days in R-10 medium (RPMI supplemented with 10% FBS, 100 U/mL penicillin G, 100 μL/mL streptomycin sulfate [Thermo Fisher Scientific], 1.7 mM sodium L-glutamine [Lonza] and 50 IU/mL IL-2 [R&D Systems]). On day 5, cells were collected and stained for viability using Violet LIVE/DEAD Cell Stain kit (Invitrogen) and anti-CD3-APC-H7 (clone SK7; BD Biosciences) and anti-CD8-PE (clone RPA-T8; Biolegend). Finally, cells were washed and fixed for 20 minutes at 4°C with 4% paraformaldehyde solution (PFA; Sigma-Aldrich). Multiparametric flow cytometry analyses were performed on an LRS Fortessa flow cytometer using FACS Diva software (BD Biosciences). Data were analyzed using the FlowJo 10.7.1 software (Treestar).

### HIV-specific T cell response.

PBMCs were thawed and resuspended in R-10 medium containing 10 U/mL DNase I (Roche Diagnostics) for 1 hour at 37°C. Afterward, cells were stimulated at 1 × 10^6^ cells/mL with 1 μg/mL of anti-CD28, 1 μg/mL of anti-CD49d (BD Biosciences), 10 μg/mL of brefeldin A (BFA; Sigma Chemical Company), and 0.7 μg/mL of monensin (BD Biosciences), in the absence or presence of 1 μg/mL of the overlapped HIV (Gag)-specific peptide pool for 6 hours. Cells were stained with conjugated monoclonal anti-CD107a-BV650 (clone H4A3; BD Biosciences) at the beginning of the incubation (57).

Stimulated PBMCs were washed with phosphate-buffered saline (PBS) and stained for 35 minutes at RT with LIVE/DEAD Fixable Aqua Dead Cell Stain (Life Technologies), anti-CD27-APC-H7 (clone M-T271; BD Biosciences), anti-CD14-BV510 (clone MΦP9; BD Biosciences), anti-CD19-Bv510 (clone SJ25C1; BD Biosciences), anti-CD56-BV510 (NCAM16.2; BD Biosciences), anti-CD8-PerCP-Cy5.5 (clone SK1; BD Biosciences), anti-CD45RA-PeCy7 (clone L48; BD Biosciences) and anti-CD3-BV711 (clone SP34-2; BD Biosciences). Subsequently, cells were washed and permeabilized with BD Cytofix/CytoPerm (BD Biosciences) for 45 minutes at 4°C. Afterward, cells were intracellularly stained with anti-IL-2-BV421 (clone MQ1-17H12; BD Biosciences), anti-TNFA-AF700 (clone Mab11; BD Pharmingen), anti-IFNG-APC (B27; BD Biosciences) and anti-PRF-Pe (B-D48; Biolegend) for 30 minutes at 4°C. Finally, cells were washed and fixed for 20 minutes at 4°C with 4 % PFA. Multiparametric flow cytometry analyses were performed on an LRS Fortessa flow cytometer using FACS Diva software (BD Biosciences). Data were analyzed using the FlowJo 10.7.1 software (Treestar).

### Thymic function assay.

Thymic function was measured by ddPCR, quantifying the presence of T cell receptor rearrangement excision circles (TRECs), sj-TREC and DβJβ-TRECs, from previously extracted DNA. The PCR program was run according to the manufacturer’s protocol using an annealing temperature of 59°C. The primers and probes used for sj-TREC were: DTR66 (5′-TGACATGGAGGGCTGAAC-3′), DTF7 (5′-AGGCTCTGTCTAGTGTGATAAC-3′), and SD1 probe (HEX-CACCCCTCTGTTCCCCACA-BHQ1). The primers and probes used for DβJβ-TRECs were: T3A (5′-CTTTCGATGGACCCTCACAG-3′), T3B (5′-GACAAG GCACCAGACTCACAG -3′), T3C (5′- AAGCTCTGGAAGGGAACACAG -3′), T3D (5′-CCGTTTCTCTCCCTCACACAG-3′), T3E (5′-GGGCAGAAACTGAGAAC ACAG-3′), T3F (5′-CTTGCGCCTTATGCTGCACAG-3′), T2 (5′-CCCAGGA GGAAAGAAGAGGAC-3′), and PB1 probe (6FAM-TGGGAGTTGGGACCG CCAGAGAGG-BHQ1). RPP30 was used as a housekeeping gene to normalize sj-TREC and DβJβ-TRECs copies (see section HIV-1 DNA quantification for RPP30 primers and probe sequences). Data were analyzed using Bio-Rad QuantaSoft software version 1.7.4.

### Statistics.

Nonparametric statistical analyses were performed using Statistical Package for the Social Sciences software (SPSS 22.0; SPSS Inc.), and graphs were done using GraphPad Prism version 8.4.2 (GraphPad software Inc). Differences between PCs and TCs were tested for statistical significance using Mann-Whitney U tests (2-tailed) and FDR-adjusted 2-tailed Fisher’s exact tests. Correlations between variables were assessed using the Spearman rank test. All *P* values < 0.05 were considered statistically significant. Polyfunctionality pie charts and Permutation test were done using Pestle version 1.6.2 and Spice version 6.0 (58).

### Study approval.

This study was in compliance with the local legislations and it was performed according to the ethical guidelines of the Declaration of Helsinki. The study was approved by the Ethics Committee of Virgen del Rocio University Hospital (Seville, Spain) (Code: 1594-N-17). All participants provided written informed consent.

### Data availability.

Owing to study participant confidentiality concerns, full-length viral sequencing data cannot be publicly released but will be made available to investigators upon reasonable request and after signing a data sharing agreement. Correspondence and requests for data should be addressed to ERM. Values for all data points in graphs are reported in the Supporting Data Values.

## Author contributions

All authors critically reviewed and approved the submitted version of the manuscript. LELC, ARCR, JS, RP, CGA, LMM, ARS, MF, ARJ, CRO, CHT, AR, JO, MALR, FV, CV, MC, AM, NE, MP, JP, AR and LFLC recruited the participants, provided PLWH blood samples, and analyzed data. SB, JV, MREIB, and FJO analyzed and interpreted the data. ERM, ML, XGY, and CGC designed the experiments. CGC, XL, CG, ICR, MRJL, GG, MICS, IG, and APG performed the experiments and analyzed and interpreted the data. CGC and ERM analyzed, interpreted the data and wrote the manuscript. ERM conceived the idea, designed the project together with LFLC, coordinated the project together with ML and XGY, and acquired funding for the study.

## Supplementary Material

Supplemental data

ICMJE disclosure forms

Supporting data values

## Figures and Tables

**Table 1:**
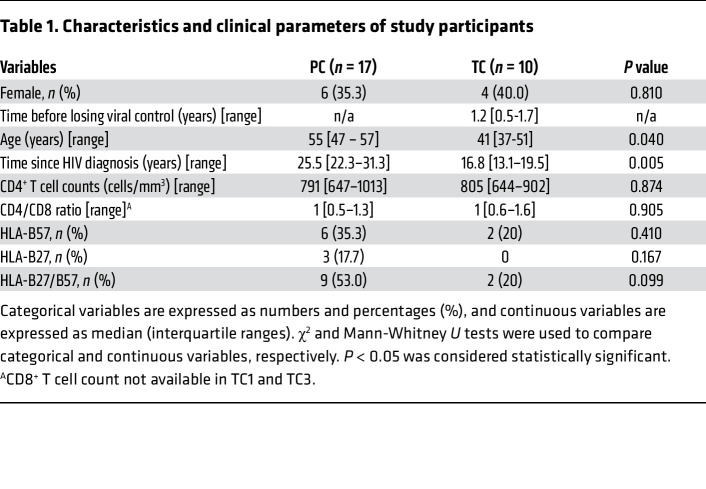
Characteristics and clinical parameters of study participants

## Version 1 02/20/2024

In-Press Preview

## Version 2 04/15/2024

Electronic publication
