# Supplementary material for: The HIV-1 reservoir landscape in persistent elite controllers and transient elite controllers
Source: J Clin Invest. 2024 Feb 20;134(8):e174215. doi: 10.1172/JCI174215 (PMC11014653; doi:10.1172/JCI174215)
Supplement: Supplemental data [file jci-134-174215-s077.pdf]

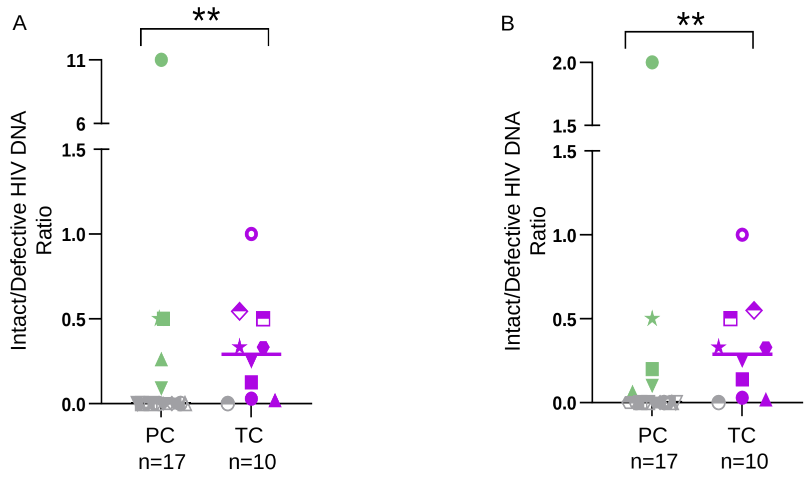

Supplemental Figure 1. Intact/Defective HIV DNA ratio. Intact/Defective HIV DNA ratio, including clonal sequences **(A)**. Intact/Defective HIV DNA ratio, excluding clonal sequences **(B)**. Grey dots represent values below the limit of detection and for this calculation has been considered as zero. Each dot represents a participant. PC and TC are represented by unique identifiers (Supplemental Table 1 and 2). Mann-Whitney U test was used to compare PC and TC participants. P value <0.05 was considered statistically significant.

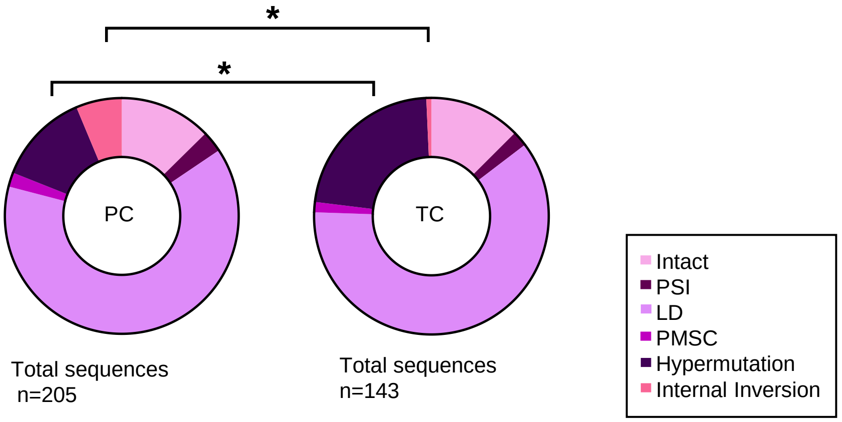

Supplemental Figure 2. Proportions of genome-proviral sequences, including clones. Intact and defective proviruses as packaging signal defect (PSI), large deletion (LD), premature stop codon (PMSC) hypermutations and internal inversion, were included. False-discovery rate (FDR)-adjusted two-tailed Fisher's exact tests were used to compare PC and TC. P value <0.05 was considered statistically significant.

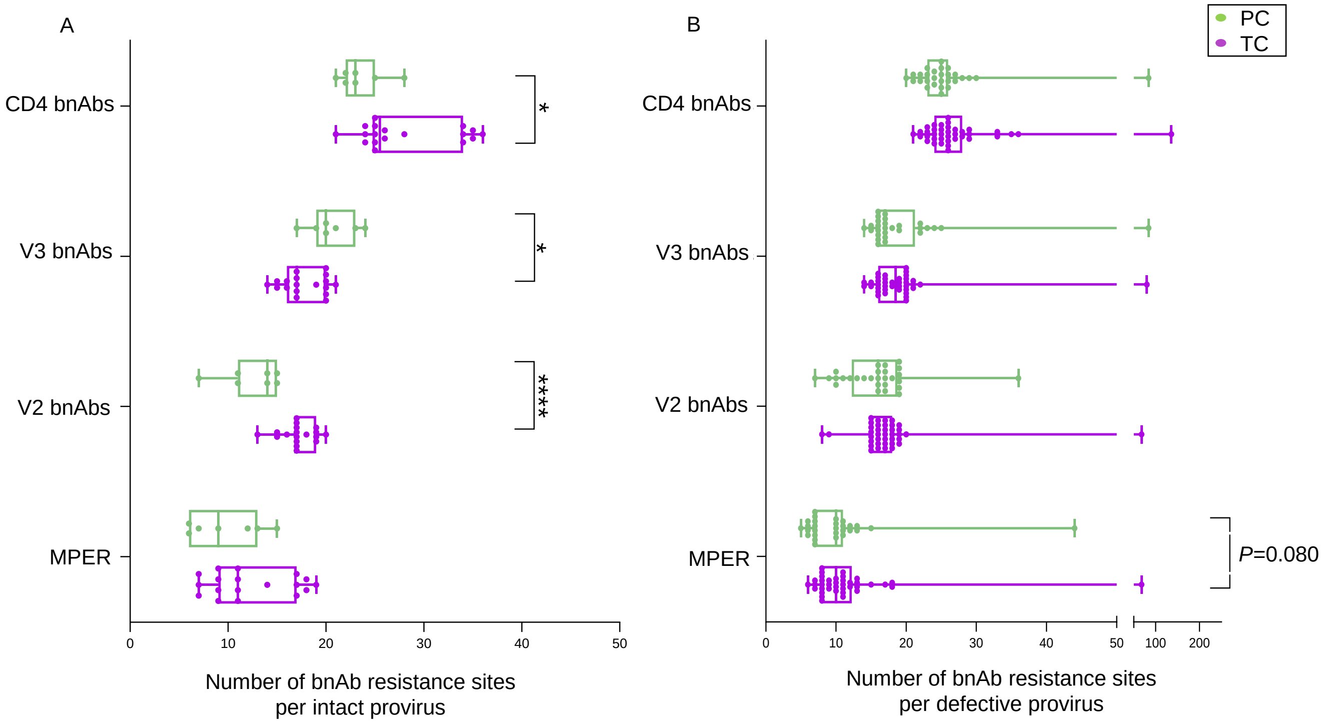

Supplemental Figure 3. Analysis of broadly-neutralizing antibody (bnAb) associated with resistance to four classes of bnAbs in intact and defective proviral sequences of PC and TC. Number of bnAb resistance sites per intact **(A)** and defective **(B)** provirus in PC and TC. Four classes of bnAbs, specific for the CD4 binding site, V3 and V2 domain and MPER region, are represented. Each dot represents an intact or defective proviral sequence. Mann-Whitney U test was used to compare PC and TC. P value <0.05 was considered statistically significant.

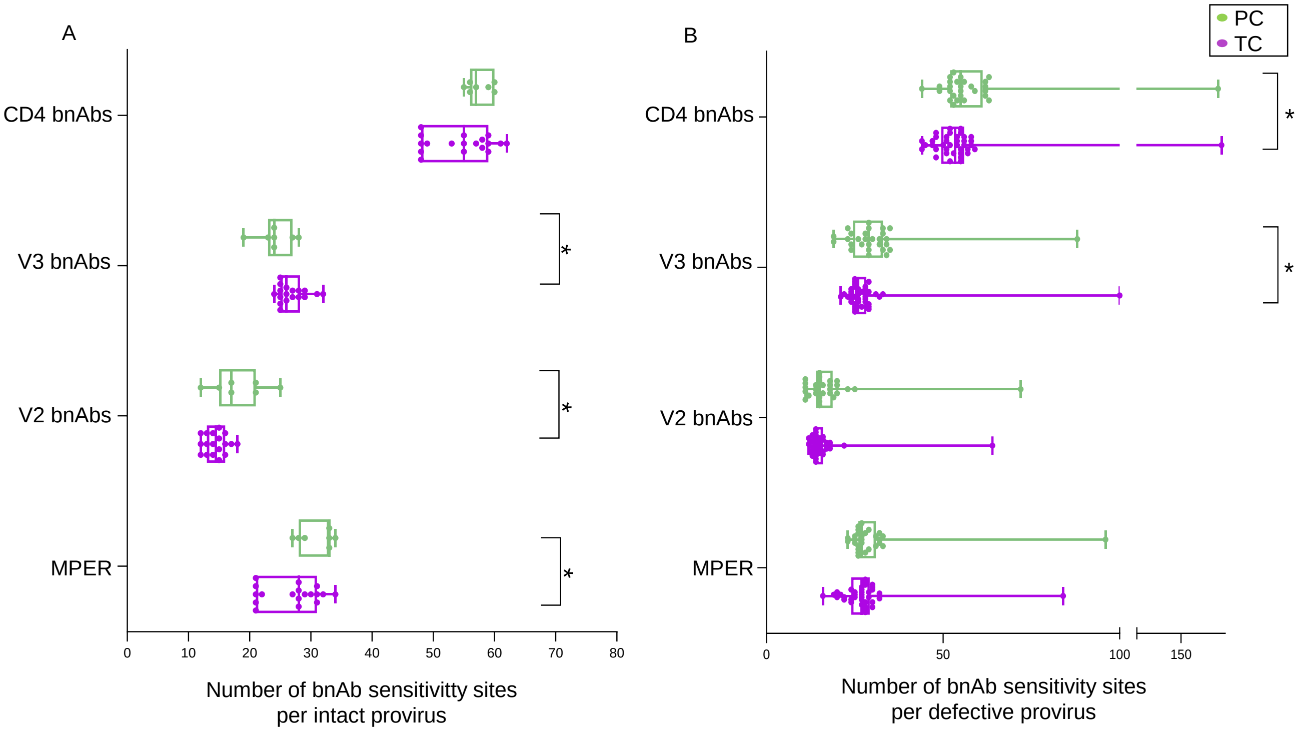

Supplemental Figure 4. Analysis of broadly-neutralizing antibody (bnAb) associated with sensitivity to four classes of bnAbs in intact and defective proviral sequences of PC and TC. Number of bnAb sensitivity sites per intact **(A)** and defective **(B)** provirus in PC and TC. Each dot represents an intact or defective proviral sequence. Mann-Whitney U test was used to compare PC and TC. P value <0.05 was considered statistically significant.

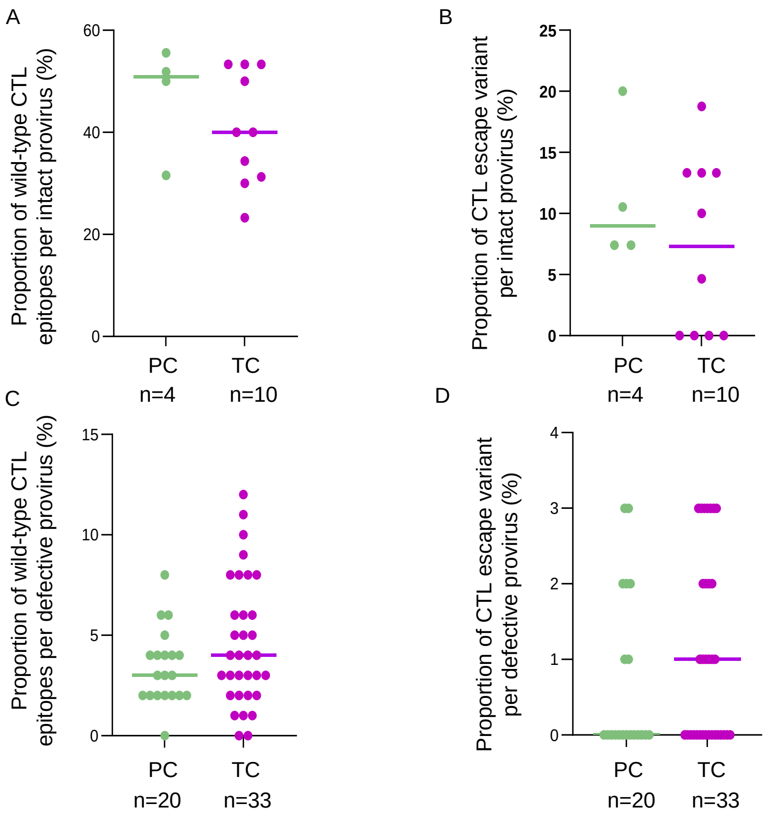

Supplemental Figure 5. Proportions of optimal CTL epitopes (restricted by autologous HLA class I alleles) with wild-type clade B consensus sequences or with previously described CTL escape mutations in PC and TC. Proportion of wild-type CTL epitopes per intact **(A)** and defective **(C)** provirus. Proportion of CTL escape variant per intact **(B)** and defective **(D)** provirus. Each dot represents an intact or defective proviral sequence. Mann-Whitney U test was used to compare PC and TC. P value <0.05 was considered statistically significant.

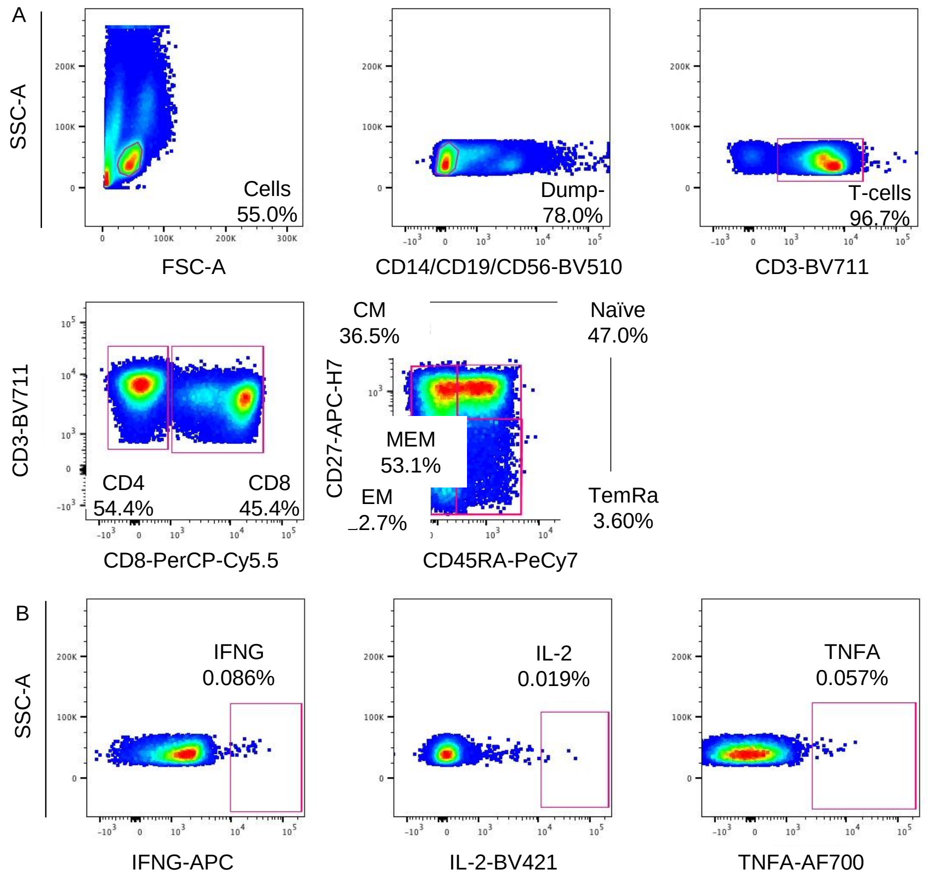

Supplemental Figure 6. Schematic diagram of the gating strategy. Phenotyping of CD4+ and CD8+ T-lymphocyte subsets, including naïve (Naïve), Memory (MEM), Central Memory (CM), Effector Memory (EM) and terminally differentiated memory (TemRa) T-cells **(A)**. Gating of intracellular cytokine production, including interferon gamma (IFNG), interleukin-2 (IL-2) and tumor necrosis factor alpha (TNFA) **(B)**.

Supplemental Table 1. Characterization of HIV-1 reservoir, from PBMCs, by FLIP-seq and MIP-seq in PC.

ID	Clade	Number of cells assayed	Total sequences	Intact sequences	Defective sequences	Identifiers
PC1	A1	5.73×10^6	48	10	38	▲
PC2	B	2.59×10^6	12	11	1	●
PC3	N.A	21.6×10^6	24	0	24	◆
PC4	N.A	9.2×10^6	12	0	12	◮
PC5	B	15.0×10^6	4	0	4	⧨
PC6	B	9.9×10^6	6	0	6	⬣
PC7	B	7.53×10^6	3	1	2	★
PC8	N.A	4.34×10^6	1	0	1	◐
PC9	N.A	13.18×10^6	1	0	1	⬣
PC10	B	1.61×10^6	19	0	19	◓
PC11	B	4.87×10^6	42	0	42	⬒
PC12	B	13.4×10^6	12	1	11	▼
PC13	N.A	2.51×10^6	1	0	1	◧
PC14	A1/G (CRF13)	2.13×10^6	9	3	6	■
PC15	N.A	11.5×10^6	9	0	9	⬣
PC16	N.A	8.12×10^6	1	0	1	⬘
PC17	N.A	4.16×10^6	1	0	1	⭘

Clades of intact HIV-1 proviral sequences, number of cells assayed and total, intact and defectives sequences accounted for by each PC. N.A.: Not available, not possible to determine through the sequencing data.

Supplemental Table 2. Characterization of HIV-1 reservoir, from PBMCs, by FLIP-seq and MIP-seq in TC.

ID	Clade	Number of cells assayed	Total sequences	Intact sequences	Defective sequences	Identifiers
TC1	B/F1 (CRF12)	4.65x10^{6}	17	6	11	⬘
TC2	B	10.47x10^{6}	15	3	12	▼
TC3	B	5.10x10^{6}	35	1	34	●
TC4	B	8.12x10^{6}	6	3	3	⭘
TC5	B	3.39x10^{6}	3	1	2	⬒
TC6	N.A.	1.33x10^{6}	2	0	2	◓
TC7	B	1.35x10^{6}	4	1	3	⬣
TC8	B	6.44x10^{6}	9	1	8	■
TC9	B	5.88x10^{6}	4	1	3	★
TC10	B	6.39x10^{6}	48	1	47	▲

Clades of intact HIV-1 proviral sequences, number of cells assayed and total, intact and defectives sequences accounted for by each TC. N.A.: Not available, not possible to determine through the sequencing data.

Supplemental Table 3. Characterization of HIV-1 reservoir, from PBMCs, by ISLA in PC and TC. Intact HIV-1 integrations sites.

ID	Type of sequence	Integration site
PC1	Intact	Chr.12: 133008698<br><br>ZNF26*
PC1	Intact	Chr.15: 19309553<br><br>Centromeric satellite DNA*
PC2	Intact	Chr.19: 53083539<br><br>ZNF160*
PC2	Intact	Chr.19: 37717470<br><br>ZNF607
TC1	Intact	Chr.11: 118451980<br><br>KMT2A
TC2	Intact	Chr.5: 50724233<br><br>PARP8

Coordinates of chromosomal integration sites and corresponding gene name of intact proviruses from PC1 and PC2; and TC1 and TC2. *Identical integration sites in several clonal intact proviruses.
